# Enantiomer-Specific Nucleation Phase Selection under Nonequilibrium Optical Trapping

**DOI:** 10.1021/acs.jpclett.6c00978

**Published:** 2026-04-27

**Authors:** Wen-Chi Wang, Qing-Yu Zhang, Kazuki Okano, Hiroshi Y. Yoshikawa, Teruki Sugiyama

**Affiliations:** † Department of Applied Chemistry and Center for Emergent Functional Matter Science, 34914 National Yang Ming Chiao Tung University, Hsinchu 300093, Taiwan; ‡ Department of Chemistry, Saitama University, Shimo-okubo 255, Sakura-ku, Saitama 338-8570, Japan; § Graduate School of Engineering, 13013 The University of Osaka, 2-1 Yamada-oka, Suita, Osaka 565-0871, Japan; ∥ Division of Materials Science, Graduate School of Science and Technology, Nara Institute of Science and Technology, Ikoma 630-0192, Japan

## Abstract

Precise control of nucleation pathways under nonequilibrium optical trapping conditions remains a fundamental challenge. Here, we report an enantiomer-specific reversal in phase selection during the optical trapping-induced crystallization of binary systems containing acetaminophen and either l- or d-phenylalanine. Switching the handedness of circularly polarized light reverses the dominant product between a thermodynamically stable cocrystal and a metastable phenylalanine phase. *In situ* Raman spectroscopy reveals constant local stoichiometry during irradiation, indicating the absence of a macroscopic polarization-induced concentration gradient. Instead, the results are consistent with a proposed mechanism where phase selection is driven by a polarization-dependent kinetic bias under strongly nonequilibrium conditions. We propose that this bias originates from subtle differences in the residence dynamics of transient nanoscale clusters within the optical trapping field, which are statistically amplified over time. These findings highlight a sophisticated kinetic route for controlling crystallization beyond conventional thermodynamic strategies.

## Graphical abstract

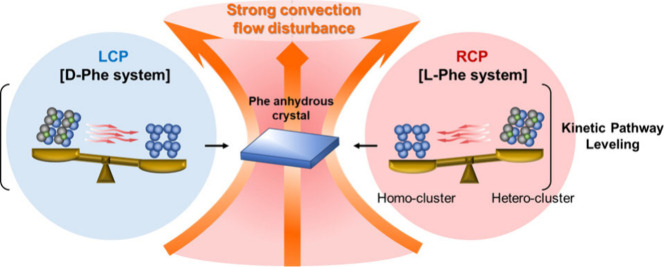

Cocrystallization has become an important strategy for controlling the physicochemical properties of molecular solids, particularly in pharmaceutical systems where crystal form can influence solubility, stability, and bioavailability.^1–3^ Cocrystals are multicomponent solids formed by incorporating coformers, such as amino acids, into the crystal lattice of an active pharmaceutical ingredient (API).^4,5^ While conventional methods rely on solution-based or solid-state approaches, laser-induced nucleation (LIN) has recently attracted attention as a means to accelerate and control crystallization processes.^6–9^ Among these approaches, optical trapping-induced crystallization (OTIC) utilizes the optical gradient force of a focused laser beam to locally concentrate molecules and induce nucleation within a confined volume.^10–12^ However, a fundamental question in OTIC remains unresolved: does the intense optical field simply act as a “concentration tweezer” that increases local supersaturation (a thermodynamic effect), or can it actively manipulate molecular assembly and nucleation pathways (a kinetic effect)? This question becomes particularly important for chiral molecular systems under high-power irradiation. Localized heating inherent to optical trapping can generate strong thermal convection, producing a dynamic environment in which fragile nucleation precursors may be destabilized.^13,14^ Under such conditions, the interplay between light chirality (circular polarization) and molecular chirality could influence the survival probability of specific molecular clusters.^15,16^ In this study, we investigate the OTIC in binary systems of acetaminophen (Ace) with l-phenylalanine (l-Phe) and its enantiomer d-phenylalanine (d-Phe) under continuous-wave (CW) laser irradiation with controlled polarization modes. We demonstrate that circularly polarized optical trapping selectively biases the crystallization pathway, leading to a notable reversal: right-handed circularly polarized light (RCP) favors Phe anhydrous crystals in the l-Phe system, whereas left-handed circularly polarized light (LCP) favors them in the d-Phe system. These results provide evidence of a proposed mechanism in which chiral optical fields act as a kinetic filter, governing the survival of nanoscale clusters and ultimately determining the macroscopic crystal form.

Building on our previous work demonstrating the utility of OTIC for cocrystallization,^17^ upon CW laser irradiation of a saturated 1:1 molar mixture of Ace and l-Phe in D_2_O, one single crystal was formed at the laser focus within 20–30 min and continued to grow while optically trapped. Figure 1 shows representative transmission images of the crystallization process under LCP laser irradiation at 1.3 W. Prior to irradiation, no solid particles were visible in the observation region (Figure 1a). During irradiation, crystals appeared at the focal point and gradually increased in size (Figure 1b–e). Similar crystallization behavior was observed for the Ace/d-Phe system. Notably, two distinct crystal morphologies were observed: needle-like (Figure 1c) and plate-like (Figure 1e). *In situ* Raman spectroscopy identified the needle-like crystals as the hemihydrate cocrystal and the plate-like crystals as anhydrous phenylalanine (Figure S4). To clarify the thermodynamic stability of these two phases, we conducted a slurry conversion experiment where an Ace/l-Phe solution containing both cocrystal and anhydrous l-Phe seeds was stirred at 25–40 °C. This temperature range was selected to cover the estimated local temperature rise induced by laser irradiation (approximately 3.0 K at 1.6 W, SI 2). The results showed that Phe anhydrous crystals dissolved while cocrystals grew, confirming that the cocrystal remains the thermodynamically stable phase even at the elevated temperatures of the laser focus, whereas the Phe anhydrous crystal is a metastable crystal form under the experimental conditions (SI 3). Thus, the formation of anhydrous Phe by the OTIC represents a kinetic selection of a metastable crystal form, despite the thermodynamic preference for the cocrystal.

**Figure 1:**
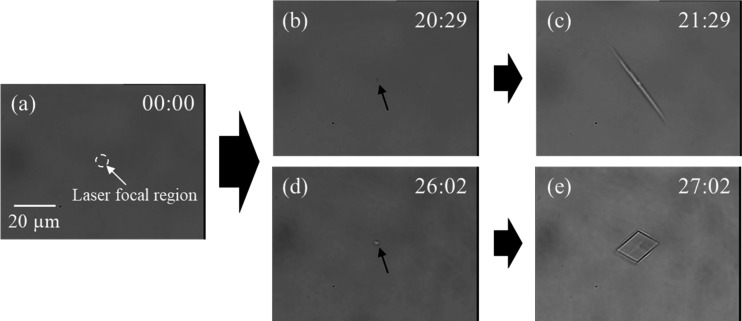
Representative transmission images illustrating crystal formation dynamics under laser irradiation. (a) Initial state. No solid is visible in the laser focal region, as indicated by the white circle. A 20 μm scale bar is shown. (b) and (c) show the formation and growth of needle-like Ace·l-Phe·0.5 D_2_O crystals. (d) and (e) show the formation and growth of plate-like l-Phe·0 D_2_O crystals. The numerical labels in the images indicate time elapsed in minutes: seconds format. All experiments were conducted with a 1.3 W LCP laser on a 1:1 molar ratio D_2_O saturated solution of Ace and l-Phe.

We investigated the effect of laser polarization on phase selection at a laser power of 1.3 W using three polarization modes (LCP, LP, and RCP; 20 trials each) in two independent saturated D_2_O solutions (Ace/l-Phe and Ace/d-Phe) at a 1:1 molar ratio (*SS* = 1.0). For the l-Phe system (Figure 2a), Fisher’s exact test comparing LCP and RCP indicated a significant polarization dependence (*p* = 0.041, SI 4). While LCP produced an approximately 50:50 distribution of cocrystals and metastable anhydrous l-Phe, RCP irradiation strongly favored the metastable phase (85%). We next examined the enantiomeric d-Phe system (Figure 2b). Interestingly, a statistically significant reversal was observed (*p* = 0.041): LCP favored metastable anhydrous d-Phe (85%), whereas RCP yielded an approximately equal distribution. This mirror-image symmetry (l-Phe/RCP and d-Phe/LCP) strongly indicates a chiral interaction between the handedness of circularly polarized light and molecular chirality. In separate control experiments using pure Ace solutions, no crystallization occurred under identical laser irradiation conditions. This indicates that the observed phase selection is essentially a competitive process between the cocrystal and Phe anhydrous crystal pathways, as independent Ace nucleation is intrinsically unfavorable under these OTIC conditions.

**Figure 2:**
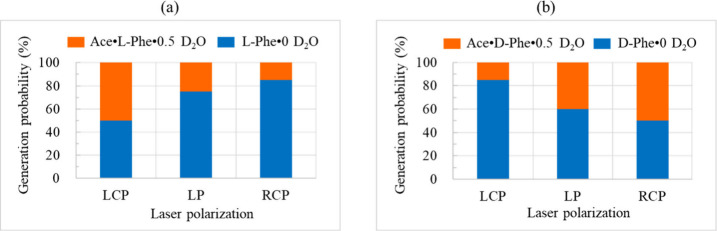
Bar graphs showing the laser polarization dependence of crystal generation probability (%) for (a) the l-Phe system (Ace/l-Phe) and (b) the d-Phe system (Ace/d-Phe). The orange bars represent the hemihydrate cocrystals (Ace·Phe·0.5 D_2_O), and the blue bars represent the phenylalanine anhydrous crystals (Phe·0 D_2_O). All experiments were conducted with a laser power of 1.3 W in D_2_O saturated solutions (Ace/l-Phe and Ace/d-Phe) at a 1:1 molar ratio. Twenty experiments were performed for each polarization condition.

To determine if this selectivity was driven by selective concentration (e.g., preferential trapping of Phe over Ace), we monitored the time evolution of the local supersaturation ratio (*SS*_Ace_/*SS*_Phe_) at the laser focus using *in situ* Raman spectroscopy, as detailed in Figure 3. By establishing independent calibration curves for both Ace and Phe (Figures S6 and S7), we calculated the temporal profiles for the l-Phe system (Figure 3a,b) and the d-Phe system (Figure 3c,d) under LCP and RCP irradiation, respectively. Each data point represents the average of 3–5 samples that had not yet undergone nucleation at the respective time points; therefore, the sample size (*N*) naturally decreases over time. Importantly, comparing the conditions that induced the reversal phenomenon—specifically RCP for l-Phe (Figure 3b) and LCP for d-Phe (Figure 3c)—reveals no systematic anomaly in the concentration dynamics. While the local *SS* ratio exhibited instantaneous fluctuations between approximately 0.7 and 1.3, these variations showed no systematic correlation with the applied circular polarization (LCP vs RCP) or the final crystal form outcome. As detailed in the Supporting Information (SI 5), error propagation analysis suggests that these transient fluctuations are consistent with the dynamic experimental noise inherent to a strongly convective, nonequilibrium environment. The time-averaged stoichiometry remains essentially 1:1, and the error bars (standard deviation) in Figure 3 consistently overlap with the stoichiometric line (1.0). This statistical analysis supports the absence of a macroscopic polarization-induced concentration bias between the API and the coformer. Given the extremely high absolute *SS* values (>4.0) at the focal point, these results indicate that Ace and Phe are trapped synchronously, maintaining the 1:1 molar stoichiometry (Tables S3, S4, and S5). Consequently, the observed enantiomer-specific crystal selection is unlikely to be attributed to a polarization-induced imbalance in the thermodynamic concentration. While our Raman measurements reflect the ensemble average within the focal volume, macroscopic phase selection is governed by the stochastic survival of nanoscopic clusters. Even if these clusters undergo localized structural fluctuations or growth immediately preceding nucleation, such transient events remain below the detection limit of bulk concentration monitoring. Therefore, the observed stoichiometry supports the hypothesis that the optical field acts as a “kinetic filter” by biasing cluster dynamics rather than inducing a macroscopic thermodynamic shift.

**Figure 3:**
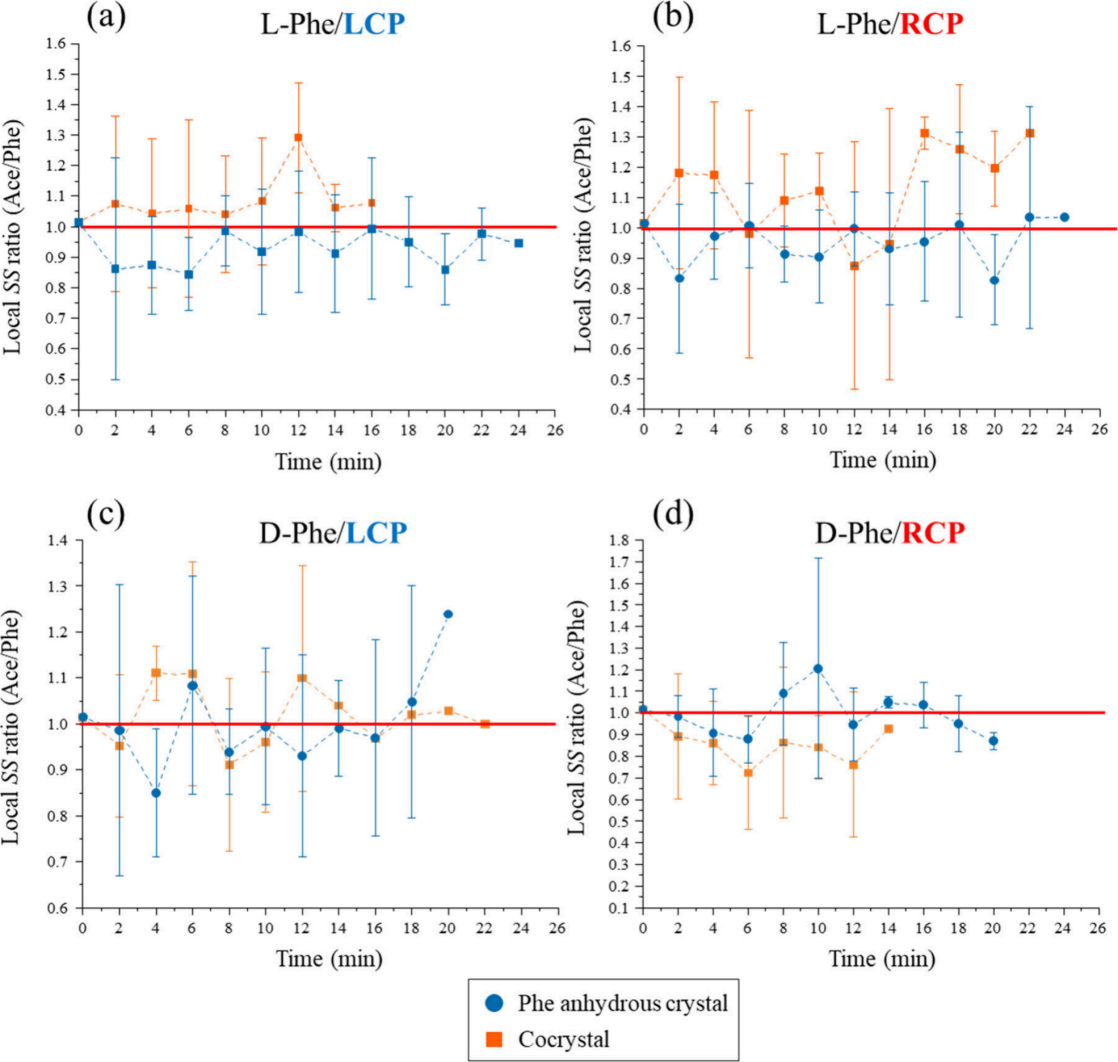
Time evolution of the local supersaturation ratio (*SS* ratio = *SS*_Ace_/*SS*_Phe_) at the laser focus for (a),(b) l-Phe system and (c),(d) d-Phe system under LCP and RCP irradiation (1.3 W). The red solid line indicates an *SS* ratio of 1.0, representing the stoichiometric ratio of the initial solution. Error bars represent the standard deviation calculated from *N* = 3–5 independent experiments. The data show that the composition ratio fluctuates around the stoichiometric value of 1.0 regardless of the polarization mode or the resultant crystal species. Crucially, the error bars largely overlap with the stoichiometric line, indicating that no statistically significant polarization-induced selective concentration occurs during the process.

Having ruled out macroscopic concentration effects, we interpret this phase selection within a two-tier nonequilibrium kinetic framework (Figure 4). In the first tier, the intense convective flow inherently generated at the laser focus acts as a “dynamic disturbance” (Figure 4a). The formation of the stable cocrystal requires a complex heteroassociation of Ace and Phe molecules (Ace-Phe-Ace···). As a general conceptual explanation based on assembly complexity, we hypothesize that such an intricate multicomponent assembly is entropically demanding and expected to be more susceptible to stochastic disruption under a strong convective flow.^18,19^ While further system-specific investigations would be required to quantify these stability differences, penalizing this complex pathway suggests that the system dynamically favors the structurally simpler homoassociation of anhydrous Phe, consistent with Ostwald’s step rule.^20,21^ This “stochastic balancing” effectively neutralizes the intrinsic thermodynamic advantage of the cocrystal phase, rendering the macroscopic outcome hypersensitive to subsequent kinetic biases.

**Figure 4:**
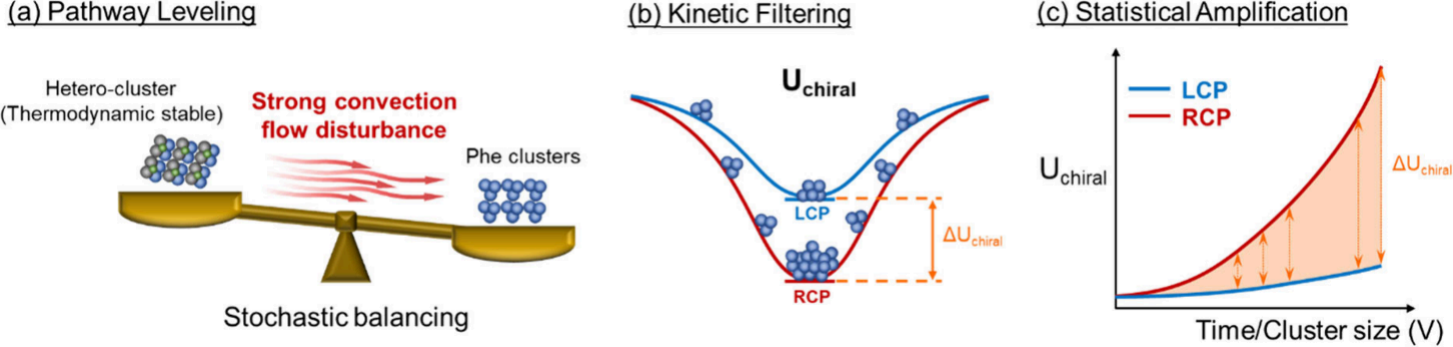
Conceptual framework for the polarization-dependent kinetic bias under nonequilibrium optical trapping. (a) Pathway Leveling: Strong laser-induced convection acts as a dynamic disturbance, disrupting the complex multicomponent assembly and leading to a stochastically balanced competition between pathways. (b) Kinetic Filtering: Circularly polarized light introduces a small, handedness-dependent chiral bias Δ*U*_*chiral*_ in the optical trapping potential. (c) Statistical Amplification: As clusters grow, their residence time in the focal region progressively increases. This enables the statistical amplification of the initial sub-*k*_*B*_ *T* bias, ultimately dictating the macroscopic phase selection.

In the second tier, within this dynamically leveled environment, the chiral optical field introduces a subtle handedness-dependent bias into the residence time of nanoscale clusters (Figure 4b). This bias originates from the intrinsic chirality of the constituent molecules. While direct measurement of circular birefringence at 1064 nm is experimentally challenging, the optical rotatory dispersion (ORD) of l-Phe is expected to extend as a normal dispersion tail toward the near-infrared region without a sign change.^22,23^ Consequently, the refractive index for right-handed circularly polarized light (*n*_*RCP*_) should remain slightly higher than that for left-handed circularly polarized light (*n*_*LCP*_) for levorotatory l-Phe. This circular birefringence generates a slightly deeper chiral trapping potential (*U*_*chiral*_) for l-Phe clusters under RCP irradiation, with the reverse occurring for d-Phe. It is also important to consider whether other polarization-dependent factors, such as local heating, interfacial hydrodynamics, or focal field structure, could contribute to the observed enantioselectivity. However, any purely mechanical or thermal effects arising from objective-induced focal field asymmetry would likely affect both the RCP and LCP pathways similarly. Given that the crystal handedness reverses with the handedness of the circular polarization, such nonchiral factors are unlikely to be the dominant contributors to the observed bias. Furthermore, numerical estimations of the local temperature rise (estimated to be approximately 3.0 K at the maximum laser power of 1.6 W, see SI 2) and induced fluid velocity suggest that these effects are insufficient to overcome thermal fluctuations at the scale of prenucleation clusters. Additionally, while chiral impurities could, in principle, lead to polarization-dependent heating via circular dichroism, the high purity of our reagents ensures that any resulting temperature differential is negligible. Therefore, we consider the residence-time amplification model, wherein the chiral trapping potential directly modulates the survival probability of nanoscopic clusters through a scaling effect, as the most plausible explanation for the observed results.

Although this initial potential difference (*ΔU*_*chiral*_) is extremely small at the molecular scale, estimated to be 10^–7^ *k*_*B*_ based on the typical ratio of circular birefringencet for amino acids extrapolated to 1064 nm, it scales cumulatively with the number of constituent molecules during cluster growth. As laser irradiation proceeds, clusters residing in the focal region continuously incorporate solutes and grow in size. Because the optical trapping potential *U* is fundamentally volume-dependent (*U* ∝ *V*), cluster growth leads to a self-amplifying expansion of *ΔU*_*chiral*_. Following the principles of Kramers’ escape rate theory,^24^ the residence time τ of a cluster in the focal region can be expressed as τ­(*V*) ∝ exp­(|*U*(*V*)|/*k*_*B*_ *T*). Under our nonequilibrium conditions where the nucleation rate *J* is small, the nucleation probability *P* during a single residence event scales as *P* ≈ *J*τ, indicating that the nucleation outcome is directly biased by the residence dynamics. Thus, the ratio of nucleation probabilities for the two polarization states follows *P*_+_/*P*_–_ ≈ exp­(*ΔU*_*chiral*_/(*k*_*B*_ *T*)). Although these nanoscopic clusters remain below the diffraction limit and are not directly visible, previous studies on related amino acid systems have shown that optical trapping can facilitate cluster growth into the several-hundred-nanometer range. For instance, dynamic light scattering studies on laser-induced phase separation of glycine have confirmed the formation and concentration of large nanodroplets around the focal point.^25^ For a 100 nm cluster, the number of constituent molecules *N* reaches ∼2.5 × 10^6^, amplifying the initial bias to ∼0.25*k*_*B*_ *T*. This cumulative increase in *ΔU*_*chiral*_ effectively biases the survival probability of specific clusters against thermal fluctuations (Figure 4c). The detailed mathematical derivations supporting this proposed residence-time amplification model are provided in the Supporting Information (SI 6).

The distinction between this proposed “residence time amplification model” and the conventional “optical concentration model” is important. In many previous studies on optical trapping-induced crystallization (OTIC), crystal formation has been attributed simply to a local increase in supersaturation. However, our *in situ* Raman analysis (Figure 3) demonstrates that macroscopic phase selection occurs without significant changes in the local solute concentration ratio. Rather than inducing a thermodynamic shift in composition or a direct lowering of the nucleation barrier, the optical field acts as a “kinetic filter” within the vigorously fluctuating fluid environment. The survival probability of a specific cluster pathway does not depend on a single nucleation event but rather on continuous success in a “competition for residence time”, which is coupled with cluster size growth and the accompanying amplification of the *ΔU*_*chiral*_. Through this statistical leverage, the initial sub-*k*_*B*_ *T* chiral bias breaks the symmetry of competing pathways, offering a consistent physical explanation for the complete reversal in phase selection probabilities under RCP/LCP observed macroscopically for l-Phe and d-Phe in Figure 2.

In conclusion, our results provide evidence that intense laser-induced convection reconstructs the crystallization energy landscape, dynamically disrupting the formation pathway of the thermodynamically stable cocrystal phases in binary l-Phe/Ace and d-Phe/Ace systems. Under this nonequilibrium environment, the chiral optical field is proposed to act as a dynamic “kinetic filter”. It biases macroscopic phase selection not through static concentration effects but likely by amplifying microscopic, sub-*k*_*B*_ *T* chiral biases via the residence time of growing clusters. These findings offer a new conceptual framework for linking microscopic chiral fluctuations to macroscopic crystallization outcomes, presenting a sophisticated kinetic strategy for phase control in multicomponent molecular systems.

Experiments were conducted on two independent binary systems: D_2_O saturated solutions containing either acetaminophen (Ace) and l-phenylalanine (l-Phe) or Ace and d-phenylalanine (d-Phe) at a 1:1 molar ratio (chemical structures in Figure S1). A continuous-wave (CW) Nd:YVO_4_ laser (λ = 1064 nm) was focused at the air/solution interface through an objective lens (60×, NA = 0.90) to induce crystallization (optical setup in Figure S2). The solution was sealed in a custom-made glass cell (Figure S3). Laser power was set to 1.3 W, and polarization (LP, LCP, and RCP) was controlled using wave plates. *In situ* Raman spectroscopy (532 nm excitation) was performed simultaneously to identify crystal forms and quantify local supersaturation (*SS*) using calibration curves (Figures S6 and S7). Crystal structures were confirmed by single-crystal X-ray diffraction (Figure S9). Detailed experimental procedures, including sample preparation, optical setup, and error analysis, are provided in the Supporting Information.

## Supplementary Material



## Notes

**Text Sections:** SI 1: Materials and Methods; SI 2: Estimation of Local Temperature Elevation at the Laser Focus; SI 3: Thermodynamic Stability Assessment (Slurry Conversion Experiment); SI 4: Statistical Evaluation of Polarization Dependence (Fisher’s Exact Test); SI 5: Error Propagation Analysis for Local Supersaturation Ratio (*SS* Ratio); SI 6: Residence-Time Amplification Model. **Figures:** Figure S1: Chemical Structures of Acetaminophen and Phenylalanine; Figure S2: Optical Setup Schematic; Figure S3: Custom-Made Sample Container; Figure S4: Raman Spectra Comparison; Figure S5: Thermodynamic Stability Assessment; Figure S6: Calibration Curve for l-Phenylalanine; Figure S7: Calibration Curves for Acetaminophen and l-Phenylalanine Used for *SS* Ratio Calculation; Figure S8: Transmittance of Solutions; Figure S9: Single-Crystal X-ray Structure of Hemihydrate Cocrystal. **Tables:** Table S1: Crystallographic Data; Table S2: Absorption Coefficients and Temperature Elevation Coefficients; Table S3: Average Achieved *SS* values (l-Phe); Table S4: Average Achieved *SS* values (d-Phe); Table S5: Average Achieved *SS* Ratio Immediately Preceding Nucleation. (PDF)
