# Supplementary material for: Enantiomer-Specific Nucleation Phase Selection under Nonequilibrium Optical Trapping
Source: J Phys Chem Lett. 2026 Apr 27;17(18):5303–8. doi: 10.1021/acs.jpclett.6c00978 (PMC13158987; doi:10.1021/acs.jpclett.6c00978)
Supplement: Supplementary file 1 [file jz6c00978_si_001.pdf]

Supporting Information

Enantiomer-Specific Nucleation Phase Selection under Non-equilibrium Optical Trapping

*Wen-Chi Wang,[1] Qing-Yu Zhang,[1] Kazuki Okano,[1,2] Hiroshi Y. Yoshikawa,[2,3] and Teruki Sugiyama[1,4]**

[1]Department of Applied Chemistry and Center for Emergent Functional Matter Science, National Yang Ming Chiao Tung University, Hsinchu 300093, Taiwan

[2]Department of Chemistry, Saitama University, Shimo-okubo 255, Sakura-ku, Saitama 338-8570, Japan

[3]Graduate School of Engineering, The University of Osaka, 2-1 Yamada-oka, Suita, Osaka 565-0871, Japan

[4]Division of Materials Science, Graduate School of Science and Technology, Nara Institute of Science and Technology, Ikoma 630-0192, Japan

Table of Contents

SI 1. Materials and Methods S3

SI 2. Estimation of Local Temperature Elevation at the Laser Focus S5

SI 3. Thermodynamic Stability Assessment (Slurry Conversion Experiment) S5

SI 4. Statistical Evaluation of Polarization Dependence (Fisher's Exact Test) S6

SI 5. Error Propagation Analysis for Local Supersaturation Ratio (*SS* Ratio) S6

SI 6. Residence-Time Amplification Model S7

Figures

Figure S1. Chemical Structures of Acetaminophen and Phenylalanine S10

Figure S2. Optical Setup Schematic S10

Figure S3. Custom-Made Sample Container S11

Figure S4. Raman Spectra Comparison S11

Figure S5. Thermodynamic Stability Assessment S12

Figure S6. Calibration Curve for L-Phenylalanine S12

Figure S7. Calibration Curves for Acetaminophen and L-Phenylalanine Used for *SS* Ratio Calculation S13

Figure S8. Transmittance of Solutions S13

Figure S9. Single-Crystal X-ray Structure of Hemihydrate Cocrystal S14

Tables

Table S1. Crystallographic Data S15

Table S2. Absorption Coefficients and Temperature Elevation Coefficients S16

Table S3. Average Achieved *SS* values (L-Phe) S16

Table S4. Average Achieved *SS* values (D-Phe) S17

Table S5. Average Achieved *SS* Ratio Immediately Preceding Nucleation S18

References S19

SI 1. Materials and Methods

Sample Preparation. Acetaminophen (Ace, ≥99.0%) and L-phenylalanine (L-Phe, ≥98.5%) were purchased from Sigma-Aldrich and used without further purification. D-phenylalanine (D-Phe, ≥98%) was obtained from Sigma-Aldrich and used without further purification. The chemical structures of Ace, L-Phe, and D-Phe are shown in Figure S1. For target solutions, saturated heavy water (D_2O) cosolutions containing Ace and L-Phe, or Ace and D-Phe, at a 1:1 molar ratio were prepared (supersaturation SS = 1.0, where $SS = C/C_0$, with C representing the actual concentration of the cocrystal and C_0 representing the saturated concentration of the cocrystal). D_2O was used as the solvent to suppress local temperature increases at the laser focus (SI 2). The preparation procedure for the Ace/L-Phe/D_2O saturated solution was as follows: First, excess amounts of Ace (20.0 mg, 0.13 M) and L-Phe (21.9 mg, 0.13 M) were added to 1.0 mL of D_2O solvent at 25 °C. This mixture was vigorously shaken in a sealed sample bottle at 60 °C for 10 hours using a temperature-controlled shaker (TAITEC, BR-21UM) to dissolve the Ace and L-Phe powders completely. After confirming dissolution, the solution was gradually cooled from 60 °C to 25 °C at 5 °C/h without shaking, until it reached room temperature. Approximately 2 hours after the start of cooling, multiple needle-like crystals precipitated within the sample vial. These crystals were identified as 1:1 hemihydrate Ace•L-Phe cocrystals (Ace•L-Phe•0.5 D_2O) by single-crystal X-ray crystallography (Figure S9). The solution containing these cocrystals was left to stand at 25 °C for approximately one week until visible crystal growth ceased, ensuring full equilibration. Subsequently, the supernatant was filtered through a 0.22 μm syringe filter to obtain the sample solution for laser experiments. Ace/D-Phe/D_2O solutions were prepared using the same procedure, with D-Phe replacing L-Phe.

Sample Cell Fabrication and Injection. 15 μL of the prepared sample solution was injected into a custom-made container (Figure S3). This container was fabricated by adhering a glass ring (thickness: 5 mm, inner diameter: 15 mm) and a cover glass (24 mm × 24 mm, thickness: 0.13-0.17 mm, NEO micro cover glass) with silicone adhesive (Shin Etsu, KE-3490). To enhance the hydrophilicity of the cover glass surface, the container was immersed in a 1% diluted Hellmanex III detergent solution (Hellma Analytics) for more than 10 hours. This ensured that upon injection, the sample solution spread immediately into a thin film with a thickness of 130–160 μm. This thin-film sample solution was necessary to meet the working distance requirements of the objective lens used in the experiments. The container was then sealed with another cover glass with a high vacuum grease (DOW CORNING CORPORATION) to produce a closed system during experiments, preventing solvent evaporation.

Optical Setup and Crystallization. A schematic diagram of the optical setup used for OTIC experiments is shown in Figure S2. An inverted microscope (Nikon, ECLIPSE Ti) was utilized in this study. A 1064 nm continuous-wave near-infrared laser beam (Nd^{3+}:YVO_4 laser, Spectra-Physics, BL-106C) was introduced into the inverted microscope after passing through two convex lenses (f_1 = 100 mm, f_2 = 200 mm), which functioned as a collimator. This laser beam was focused at the air/solution interface in the sample solution using an objective lens (Olympus, 60×, NA = 0.90). For optical alignment and solution height measurement, a 632.8 nm visible laser beam (Coherent, Melles Griot, 05-LHP-151) was also introduced into the microscope via the same

optical path as the 1064 nm trapping laser. The output power of the laser beam used in this experiment was adjusted using a half-wave plate and a beam splitter. The output power after passing through the objective lens was set to 1.0 W, 1.3 W, and 1.6 W, which approximately correspond to laser power densities of 61 MW/cm^2, 80 MW/cm^2, and 98 MW/cm^2, respectively. Laser polarization was controlled by linear polarization, left-handed circular polarization (LCP), and right-handed circular polarization (RCP) using polarizing optics (e.g., a half-wave plate). A charge-coupled device (CCD; Jai, Cv-s3200) was connected to this optical setup to observe transmission images of crystallization behavior directly. All laser experiments were conducted at 25 °C.

***In Situ* Raman Spectroscopy and Supersaturation Analysis.** The optical setup also incorporated a Raman spectroscopy system for *in situ* identification of crystals generated by laser irradiation and for monitoring concentration dynamics. A 532 nm visible laser beam (Spectra-Physics, Millennia Pro) was used as the excitation source for Raman spectroscopy (50 mW). Scattered light was collected with the same objective lens and directed to a spectrometer (ANDOR, SR-303i-A) equipped with an electron-multiplying CCD (EMCCD; ANDOR, DU401A-BVF). Local supersaturation (*SS*) at the laser focus was calculated from the characteristic peak intensity of the L-Phe solution obtained by *in situ* Raman spectroscopy. Specifically, a calibration curve was created using the 1003 cm^{-1} peak intensity, attributed to the angular deformation vibrational mode of the phenylalanine phenyl ring, to quantify the L-Phe concentration in the solution. This calibration curve was constructed from solutions of varying concentrations, measured at 25 °C (Figure S6). Using the local concentration (C) converted from Raman intensity and the saturated concentration (C_0) determined at 25 °C, an apparent local supersaturation, $SS = C/C_0$, was first calculated. This apparent *SS* value was then corrected for the local temperature increase induced by laser irradiation, which increases solute solubility. Temperature correction is essential for accurately assessing supersaturation, especially considering the polymorphic transition temperature of L-Phe crystals (approx. 37 °C).[S1] The temperature increase at the laser focus in this study was estimated to be approximately 3.0 K. It was confirmed from solubility data that this 3.0 K temperature increase causes approximately a 13% increase in the solubility of the Ace•L-Phe•0.5 D_2O cocrystal. Therefore, a correction based on the 13% increase in solubility was applied to the *SS* values obtained from the calibration curve at 25 °C to estimate the true local supersaturation. For detailed methods for estimating the temperature increase (SI 2).

Single-Crystal X-ray Diffraction (SCXRD) Analysis. Crystals spontaneously generated from 1:1 molar ratio Ace/L-Phe/D_2O or Ace/D-Phe/D_2O solutions, as well as those produced by OTIC, were mounted in glass capillaries for single-crystal X-ray analysis. Data collection was performed on a four-circle diffractometer (XtaLAB Synergy R, DW system, HyPix) using a Cu K-alpha radiation source (λ = 1.54184 Å) at temperatures of 99.98(10), 100.00(12), or 99.99(11) K. CrysAlisPro 1.171.42.96a (Rigaku OD, 2023) was used as the data collection software. The crystal orientation matrix was determined via omega-scans in accordance with the CrysAlisPro conventions. Multiple frames were acquired for each scan, with each frame corresponding to a 0.5-degree scan ranging from tens of milliseconds to tens of seconds, depending on the

measurement angle. A total of 60 or 53 measurements were performed until the hemispherical data were complete. Structure determination was carried out using the Olex2 software, with the SHELXT structure analysis program utilized in conjunction with the Intrinsic Phasing method. Structure refinement was performed using the least-squares minimization of the SHELXL package. Empirical absorption correction was applied using spherical harmonics within the SCALE3 ABSPACK scaling algorithm. All non-hydrogen atom positions were anisotropically refined and identified on clear Fourier maps, while hydrogen atoms were geometrically placed. Detailed parameters for data collection and refinement are in Table S1.

SI 2. Estimation of Local Temperature Elevation at the Laser Focus

For experiments involving optical trapping-induced crystallization, it is essential to account for the local temperature increase at the laser focus caused by photon absorption by solutes and solvents. Generally, increased temperature increases solute solubility, thereby inhibiting crystal nucleation. Moreover, temperature plays a critical role in L-Phe crystallization due to its temperature-dependent pseudopolymorphism. Therefore, estimating the temperature increase is crucial.

The temperature increase coefficient ($\Delta T/\Delta$P), representing the temperature increase per unit laser power, is proportional to the absorption coefficient (α) of the solution and inversely proportional to the thermal conductivity (λ), as shown in Eq. S1:[S2]

$$\frac{\Delta T}{\Delta P} \propto \frac{\alpha}{\lambda} \quad \text{(Eq. S1)}$$

To accurately estimate the temperature elevation of cosolutions of Ace and L-Phe based on Eq. S1, the transmittance of solvents and solutions (H_2O, D_2O, saturated Ace/L-Phe/H_2O, and saturated Ace/L-Phe/D_2O solutions) was measured at 1064 nm, the wavelength of the trapping laser (Figure S8). The absorption coefficient (α) was calculated using the Lambert-Beer law: $\alpha = \frac{ln\left(\frac{1}{T}\right)}{l}$, where T is transmittance, α is the absorption coefficient, l is the optical path length, measured using an absorption spectrophotometer (JASCO, V-670).

Figure S8 shows the transmittance of all solvents and solutions at 1064 nm as a function of the optical path length. The estimated absorption coefficients are summarized in Table S2. Assuming a thermal conductivity similar to that of H_2O, the temperature coefficient of increase was calculated. The temperature increase in the saturated Ace/L-Phe/D_2O solution is estimated to be 3.0 K at 1.6 W, the maximum power used in this study. This indicates that the local temperature increase is minimal and does not reach the phase transition point (37 °C) of L-Phe crystals.

SI 3. Thermodynamic Stability Assessment (Slurry Conversion Experiment)

To determine which crystal form corresponds to the thermodynamically stable phase and which to the metastable phase, slurry conversion experiments were conducted at 25 °C and 40 °C. This

temperature range was selected to cover the estimated local temperature rise induced by laser irradiation (approx. 3.0 K at 1.6 W, see SI 2). A saturated D_2O solution containing equimolar amounts of Ace and L-Phe was prepared. To this solution, seed crystals of both the L-Phe anhydrous crystal and the hemihydrate cocrystal of similar size were added simultaneously. The resulting suspension was kept at each temperature. In both temperature conditions, Raman spectroscopy analysis of the recovered solid phase revealed that the L-Phe anhydrous crystals had dissolved or converted, while the solid phase composed of the hemihydrate cocrystals persisted or grew (Figure S5). This observation indicates that the hemihydrate cocrystal is the thermodynamically stable phase and the L-Phe anhydrous crystal is the metastable phase under the experimental conditions. Notably, the conversion kinetics were remarkably faster at 40 °C. While the conversion took several days at 25 °C, the L-Phe anhydrous crystals completely dissolved within less than 24 hours at 40 °C. This rapid transformation indicates that the thermodynamic driving force, likely stemming from the solubility difference between the two phases, remains substantial and is kinetically accelerated at elevated temperatures.

SI 4. Statistical Evaluation of Polarization Dependence (Fisher's Exact Test)

To evaluate the statistical significance of the notable "reversal phenomenon" driven by the handedness of circularly polarized light, we performed Fisher's exact test. The null hypothesis (H_0) posited that the generation probability of the crystal forms (hemihydrate cocrystal vs. anhydrous phenylalanine) is independent of the circular polarization state (LCP vs. RCP). To directly test the enantiomeric interaction between chiral light and chiral molecules, the statistical analysis focused on a 2 × 2 contingency table comparing LCP and RCP ($N = 40$ per enantiomeric system). Fisher's exact test was chosen over Pearson's chi-squared test because it provides exact p-values for small sample sizes and 2 × 2 contingency tables.

L-Phe System: For the L-Phe system, the observed crystal distribution exhibited a significant dependence on the circular polarization. Under LCP irradiation, an equal distribution was observed (10 cocrystals, 10 anhydrous), whereas RCP irradiation strongly favored the metastable anhydrous form (3 cocrystals, 17 anhydrous). The two-tailed Fisher's exact test yielded a p-value of 0.041. Since $p < 0.05$, the null hypothesis was rejected at the 95% confidence level, indicating a statistically significant dependence of crystal selectivity on the handedness of the light.

D-Phe System: For the optical isomer D-Phe system, a striking "reversal phenomenon" in selectivity was observed. LCP irradiation strongly favored the anhydrous form (3 cocrystals, 17 anhydrous), while RCP resulted in an equal distribution (10 cocrystals, 10 anhydrous). The two-tailed Fisher's exact test for this distribution correspondingly yielded a p-value of 0.041. By employing the 2 × 2 direct comparisons of LCP and RCP, the results indicate that the crystal form selection is significantly biased ($p < 0.05$) by the interplay between the spin angular momentum of light and the molecular chirality in both enantiomeric systems. This supports the proposed polarization-dependent kinetic bias mechanism.

SI 5. Error Propagation Analysis for Local Supersaturation Ratio (*SS* Ratio)

To evaluate the reliability of the local supersaturation ratio ($SS_{\text{Ace}}/SS_{\text{Phe}}$), we performed an error propagation analysis. The SS values are derived from Raman peak intensities (I) using calibration curves with linear slopes (k), according to the relationship $SS = I/k$. Assuming the errors in intensity measurement and calibration are independent, the relative uncertainty in the SS ratio ($\frac{\delta SS_{ratio}}{SS_{ratio}}$) is estimated using the following equation:

$$\frac{\delta SS_{ratio}}{SS_{ratio}} = \sqrt{\left(\frac{\delta I_{Ace}}{I_{Ace}}\right)^2 + \left(\frac{\delta I_{Phe}}{I_{Phe}}\right)^2 + \left(\frac{\delta k_{Ace}}{k_{Ace}}\right)^2 + \left(\frac{\delta k_{Phe}}{k_{Phe}}\right)^2}$$

Based on the signal-to-noise ratio of our *in situ* Raman spectra, the intensity readout error ($\delta I/I$) is estimated to be approximately 5%. Similarly, the fitting errors of the calibration curves (Figures S6 and S7) suggest a slope uncertainty ($\delta k/k$) of approximately 5%. Substituting these values yields a baseline relative uncertainty of:

$$\sqrt{(0.05)^2 + (0.05)^2 + (0.05)^2 + (0.05)^2} \sim 0.10\ (10\ \%)$$

However, in the dynamic environment of optical trapping, additional noise sources, such as the Brownian motion of clusters within the focal volume and slight drifts within the laser focus, must be considered. Taking these factors into account, a conservative estimate of the total experimental uncertainty under active laser trapping is approximately ± 15–20%. Crucially, this variance does not solely represent instrumental error, but inherently includes physical concentration fluctuations driven by strong thermal convection and the continuous Brownian motion of nanoscopic clusters entering and exiting the focal volume. Therefore, the observed transient fluctuations of the local SS ratio (between 0.7 and 1.3, Figure 3) fall entirely within the expected margin of this dynamic environment. Furthermore, although the inherently stochastic nature of the nucleation process restricts the number of pre-nucleation samples that can be practically captured at each specific time point (N = 3–5), the consistent overlap of the standard deviations with the stoichiometric baseline (1.0) across the entire pre-irradiation period is statistically robust. This suggests that the observed variations are random dynamic fluctuations rather than the formation of a systematic, polarization-induced macroscopic concentration gradient. The time-averaged composition at the focal point remains strictly stoichiometric.

SI 6. Detailed Mathematical Derivation of the Proposed Residence-Time Amplification Model

The physical essence and the scaling analysis of the proposed residence-time amplification model are described in the Discussion section of the Main Text. This section provides the detailed mathematical derivation supporting the model.

Nucleation Probability under Finite Residence Time: Under strong convective flow, if nucleation is treated as a stochastic process with an intrinsic rate J, the probability P of nucleation during a single residence event of duration τ is expressed as

$$P = 1 - exp\,(-J\tau)$$

For $J\tau \ll 1$, which is expected under strongly nonequilibrium conditions, this simplifies to

$$P \approx J\tau$$

, indicating that nucleation probability scales linearly with residence time.

Within classical nucleation theory,

$$J = J_0 exp\,(-\frac{\Delta G^*}{k_B T})$$

where $\Delta G^*$is the intrinsic thermodynamic nucleation barrier and J_0 is a kinetic prefactor. In the present framework, the optical field does not directly modify ΔG^*.

Optical Trapping-Induced Enhancement of Residence Time:

Nanoscopic clusters in the Rayleigh regime experience an optical trapping potential:

$$\boldsymbol{U} = -\frac{1}{2}\alpha \boldsymbol{E}^2$$

where the effective polarizability α is directly proportional to the cluster volume $(\alpha \propto V)$. Under the present experimental conditions, the trapping energy remains much smaller than the thermal energy, especially during the early stages of trapping. Escape from a shallow optical potential can be approximated as a thermally activated process, following the classical Kramers' escape rate theory, yielding a residence time that scales as:[S3,S4]

$$\tau(V) \propto exp\left(\frac{|U(V)|}{k_B T}\right)$$

Importantly, as the cluster grows in size with the trapping time, the increasing cluster volume V leads to a self-amplifying deepening of the trapping potential $U(V)$, resulting in an exponential extension of the residence time τ.

.

Polarization-Dependent Bias and Kinetic Amplification:

Under circularly polarized light, the chiral optical field introduces a small chiral potential energy difference $U_{chiral,i}$ between the right- and left-handed circularly polarized light (Figure 4b). The nucleation probability $P_{\pm,i}(V)$ for a single residence event can be expressed as a function of the cluster volume V:

$$P_{\pm,i}(V) \approx J_i \cdot \tau_{\pm,i}(V)$$

where J_i is the nucleation rate for pathway *i*. Taking the ratio of nucleation probabilities for right- and left-handed circularly polarized light (P_+ and P_-):

$$\frac{P_{+,i}(V)}{P_{-,i}(V)} \approx \frac{\tau_{+,i}(V)}{\tau_{-,i}(V)} = exp\left(\frac{\Delta U_{chiral,i}(V)}{k_B T}\right)$$

For a small bias where $\Delta U_{chiral,i}(V) \ll k_B T$, the Taylor expansion yields:

$$\frac{P_{+,i}(V)}{P_{-,i}(V)} \approx 1 + \frac{\Delta U_{chiral,i}(V)}{k_B T}$$

Furthermore, since the optical trapping potential is volume-dependent, the chiral bias $\Delta U_{chiral,i}(V)$ is not a constant but scales with the cluster size. As a cluster resides in the focal region and grows through the continuous incorporation of solutes, the kinetic advantage P_+/P_- is dynamically amplified. Over the macroscopic irradiation period, this size-dependent feedback loop—where longer residence leads to larger size, which in turn further extends the residence time—statistically amplifies the initial sub-$k_B T$ bias (Figure 4c). This mechanism breaks the symmetry of competing pathways under nonequilibrium conditions, ultimately dictating the macroscopic phase selection observed in Figure 2.

To quantitatively estimate the magnitude of this amplification, we consider the scaling of the chiral bias with cluster size. Because the unique feature of this system is the enantioselective survival and nucleation of the metastable Phe anhydrous phase against the thermodynamically favored cocrystal, we focus on the Phe nanocluster as a representative case. Consistent with pre-nucleation cluster sizes reported in the literature[S5] for similar amino acid systems (e.g., glycine), where stable clusters of several hundred nanometers are observed via DLS, we adopt d = 100 nm as a typical diameter for our numerical estimation. Using the physical properties of L-Phe ($\rho \approx$ 1.34 g/cm^3, M = 165.19 g/mol), the number of constituent molecules N in a pure Phe nanocluster is calculated as follows:

$$N = \frac{\pi d^3 \rho N_A}{6M} \approx 2.5 \times 10^6$$

Given that the initial bias at the single-molecule scale is infinitesimally small (estimated as $\Delta U_{chiral,mol} \approx 10^{-7} k_B T$ under our typical optical trapping conditions), the accumulated chiral bias for a 100 nm cluster reaches:

$$\Delta U_{chiral}(100\ nm) = N \cdot \Delta U_{chiral,mol} \approx 0.25\ k_B T$$

This scaling analysis validates that the initially negligible sub- $k_B T$ bias can surpass thermal fluctuations and selectively extend the residence time of the metastable Phe cluster.

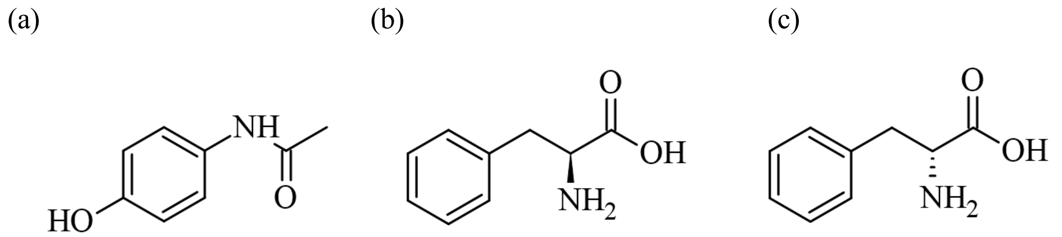

Figure S1. Chemical structure of (a) acetaminophen (Ace), (b) L-phenylalanine (L-Phe), and (c) D-phenylalanine (D-Phe).

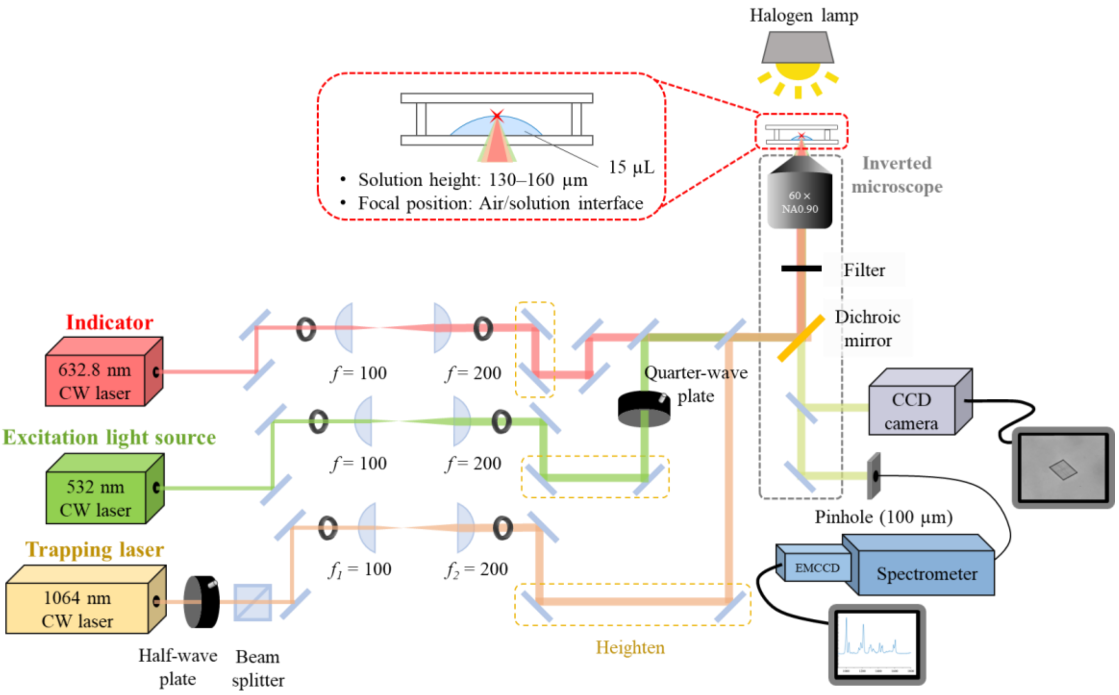

Figure S2. A schematic illustration of the optical setup used for experiments on optical trapping-induced crystallization and Raman spectroscopy. The CW Nd:YVO$_4$ laser (λ = 1064 nm) is focused at the air/solution interface of the sample solution via a 60× objective lens. A 532 nm laser is used for *in situ* Raman measurements.

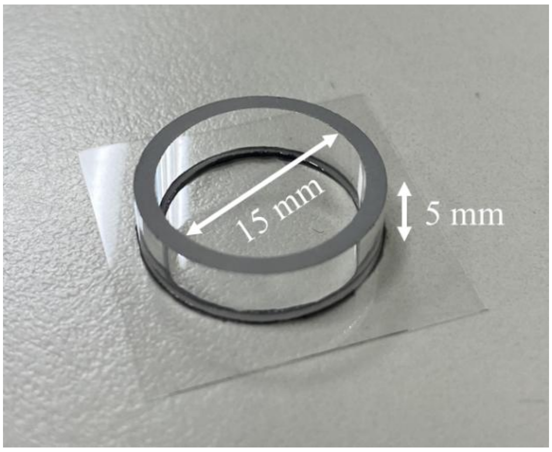

Figure S3. A photograph of a custom-made container consisting of a glass ring (thickness: 5 mm, inner diameter: 15 mm) and a cover glass.

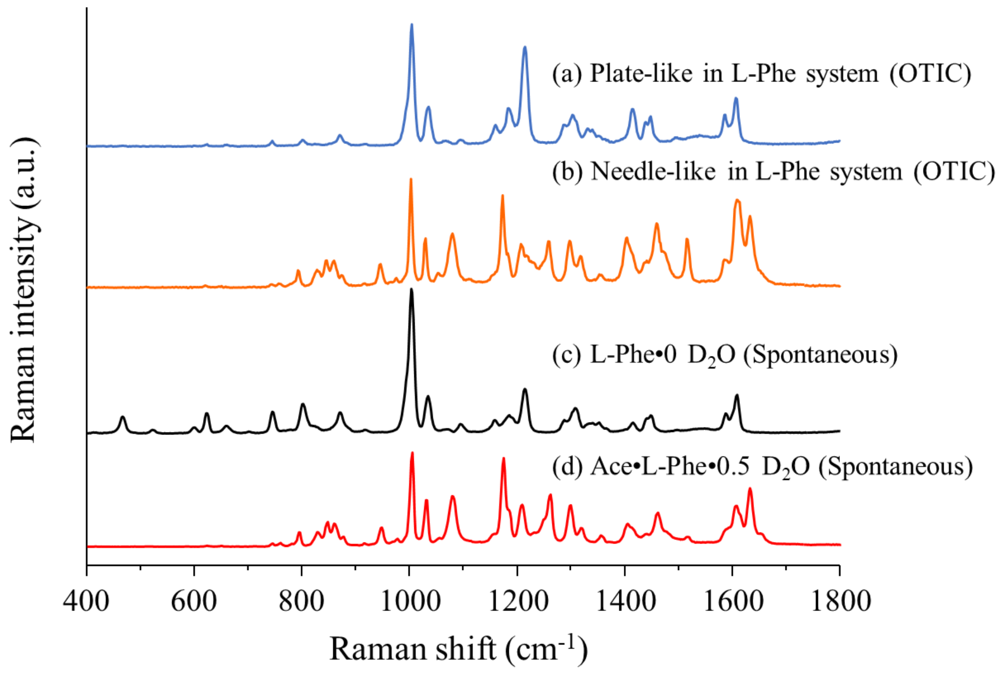

Figure S4. Raman spectra comparison of crystals generated via spontaneous nucleation and optical trapping (OTIC): (a) Plate-like crystal (blue) and (b) needle-like crystal (orange) generated by OTIC in the L-Phe system correspond to (c) L-Phe anhydrous crystal (black) and (d) 1:1 hemihydrate cocrystal of Ace and L-Phe (red), respectively. The Raman spectra of (c) and (d) were obtained from the generation of spontaneous nucleation.

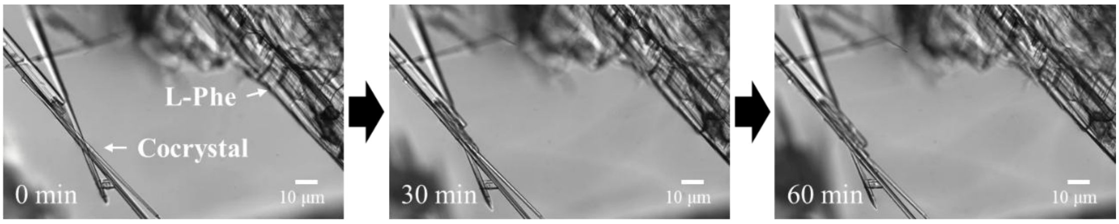

Figure S5. Thermodynamic stability assessment by slurry conversion. In a competitive suspension, L-Phe anhydrous crystals slowly dissolved while the 1:1 Ace•L-Phe hemihydrate cocrystals grew, indicating that the cocrystal is the thermodynamically stable phase, whereas the L-Phe anhydrous crystal is the metastable phase between 25 and 40 °C.

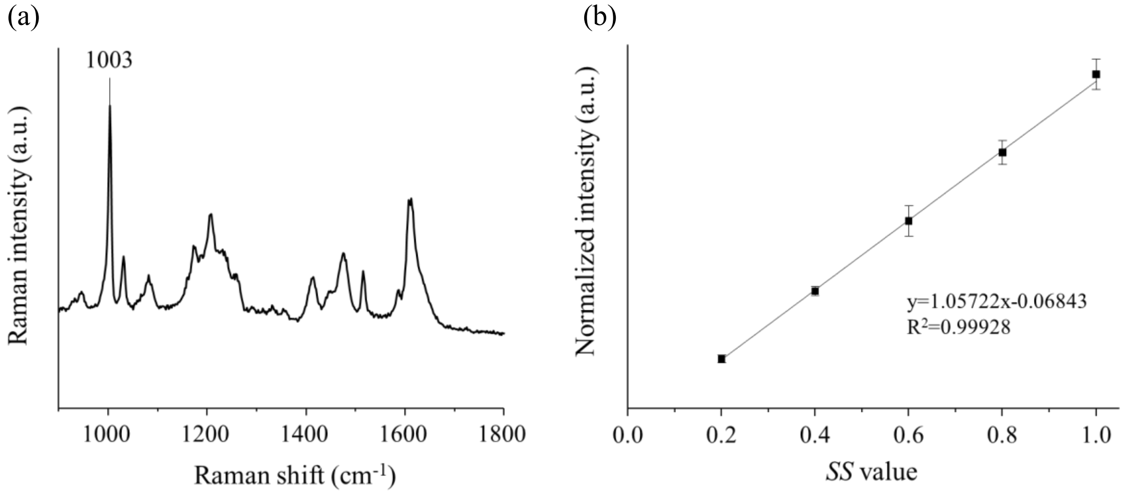

Figure S6. Calibration curve for L-Phenylalanine. (a) Raman spectrum of the solution with *SS* = 1.0 exhibiting the 1003 cm^{-1} peak. (b) The calibration curve of normalized intensity vs. *SS* value (R^2 = 0.999).

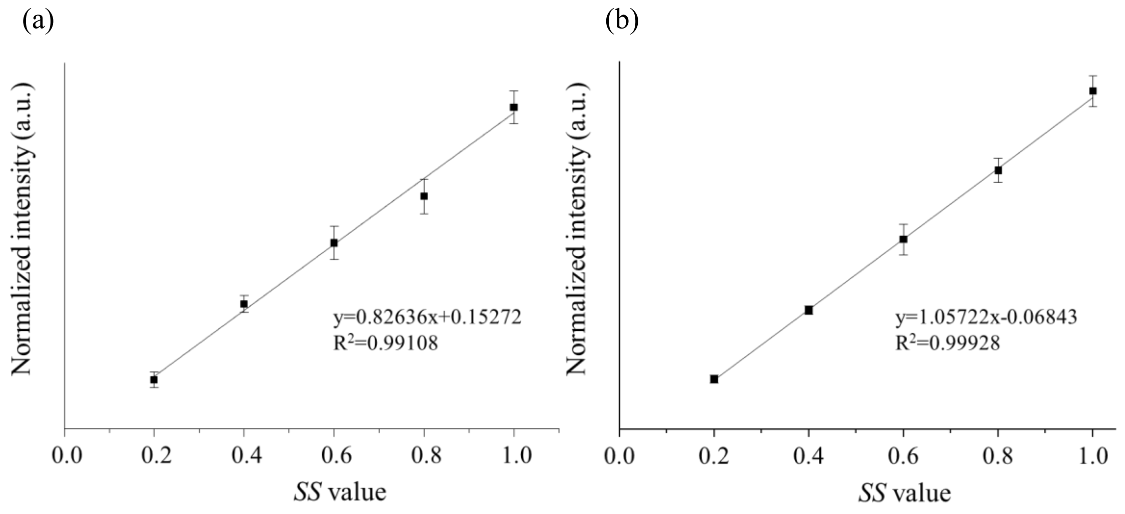

Figure S7. Calibration curves for Acetaminophen and L-Phenylalanine used for *SS* Ratio calculation. (a) Calibration curve for Ace using the 1515 cm^{-1} peak. (b) Calibration curve for L-Phe using the 1003 cm^{-1} peak.

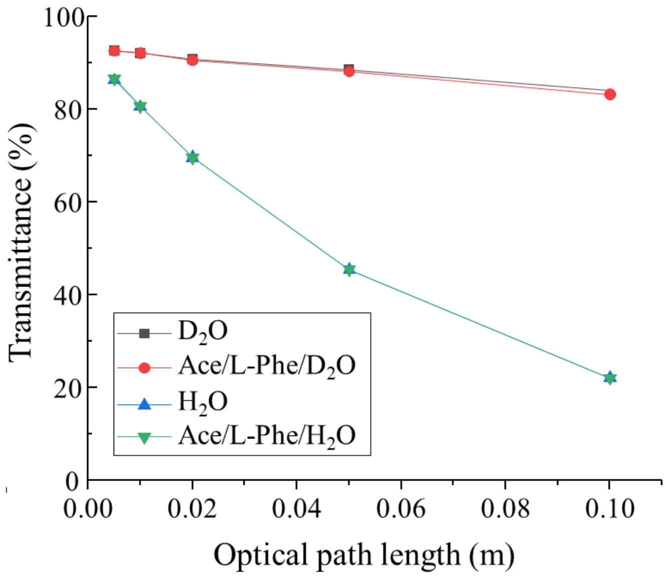

Figure S8. Transmittance of H_2O, D_2O, Ace/L-Phe/H_2O, and Ace/L-Phe/D_2O at 1064 nm as a function of optical path length.

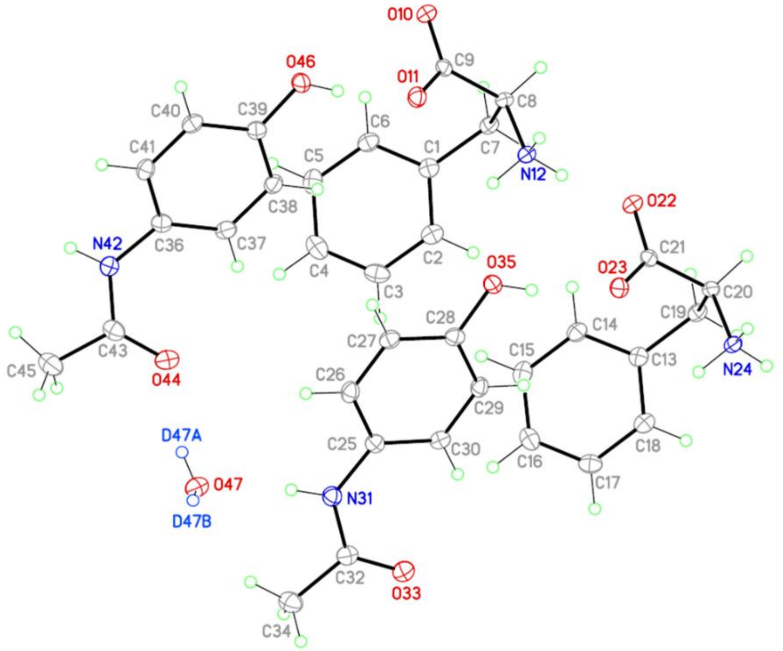

Figure S9. Single-crystal X-ray structure (ORTEP diagram) of the hemihydrate Ace•L-Phe•0.5 D_2O cocrystal. Ellipsoids are drawn at the 50% probability level.

Table S1. Crystallographic Data: Crystal data and structure refinement for 1:1 Ace•L-Phe hemihydrate crystal.

Empirical formula	$C_{34}H_{40}D_2N_4O_9$
Formula weight	652.73
Temperature/K	99.98(10)
Crystal system	monoclinic
Space group	$P2_1$
a/Å	11.61135(11)
b/Å	7.04009(7)
c/Å	20.0320(2)
α/°	90
β/°	100.7736(9)
γ/°	90
Volume/Å^3	1608.65(3)
Z	2
ρ_{calc} mg/mm^3	1.348
μ/mm^{-1}	0.810
F(000)	692.0
Crystal size/mm^3	0.16 × 0.13 × 0.04
Radiation source	Cu Kα (λ = 1.54184Å)
2Θ range for data collection/°	7.75 to 134.132

Index ranges	-13 ≤ h ≤ 13, -8 ≤ k ≤ 8, -23 ≤ l ≤ 23
Reflections collected	18858
Independent reflections	5729 [R_{int} = 0.0271]
Data/restraints/parameters	5729/1/448
Goodness-of-fit on F^2	1.043
Final R indexes [I>=2σ (I)]	R_1 = 0.0254, wR_2 = 0.0670
Final R indexes [all data]	R_1 = 0.0265, wR_2 = 0.0676
Largest diff. peak/hole / e $Å^{-3}$	0.16/-0.15
Flack parameter	0.10(13)

Table S2. Absorption coefficients of solutions and the calculated temperature elevation coefficient at the laser focus.

Sample solution	α [m^{-1}]	$\Delta T/\Delta P$ [KW^{-1}]
H_2O	14.3	～24.2
D_2O	1.007	～1.7
Ace/L-Phe/H_2O (*SS* = 1.0)	14.37	～24.4
Ace/L-Phe/D_2O (*SS* = 1.0)	1.108	～1.9

Table S3. Average achieved *SS* values and their standard deviations for each polarization condition and generated crystal species in the L-Phe system, which were calculated from (a) the calibration curve for acetaminophen and (b) the calibration curve for phenylalanine, respectively. 3–5 experiments were conducted under each condition.

(a)	**Average achieved *SS***	
**Resultant crystal**	LCP	RCP
**Ace•L-Phe•0.5 D_2O**	5.4 ± 2.9 /3	3.7 ± 3.3 /4

**L-Phe•0 D_2O**	3.1 ± 0.5 /5	5.0 ± 1.5 /5

(b)	**Average achieved *SS***	
**Resultant crystal**	LCP	RCP
**Ace•L-Phe•0.5 D_2O**	4.9 ± 2.9 /3	4.3 ± 1.5 /4
**L-Phe•0 D_2O**	3.4 ± 0.4 /5	4.9 ± 0.4 /5

Table S4. Average achieved *SS* values and their standard deviations for each polarization condition and generated crystal species in the D-Phe system, which were calculated from (a) the calibration curve for acetaminophen and (b) the calibration curve for phenylalanine, respectively. Five experiments were conducted under each condition.

(a)	**Average achieved *SS***	
**Resultant crystal**	LCP	RCP
**Ace•D-Phe•0.5 D_2O**	2.6 ± 1.0 /5	1.7 ± 0.4 /5
**D-Phe•0 D_2O**	3.6 ± 1.4 /5	4.3 ± 0.4 /5

(b)	**Average achieved *SS***	
**Resultant crystal**	LCP	RCP
**Ace•D-Phe•0.5 D_2O**	2.4 ± 1.0 /5	2.3 ± 0.6 /5
**D-Phe•0 D_2O**	3.8 ± 0.7 /5	4.5 ± 0.8 /5

Table S5. Average *SS* ratio immediately preceding nucleation for each crystal species generated under different polarization conditions.

System	Polarization	Resultant Crystal	Average *SS* Ratio
**Ace/L-Phe**	LCP	Ace•L-Phe•0.5 D_2O	1.2 ± 0.1 /3
		L-Phe•0 D_2O	0.9 ± 0.1 /5
	RCP	Ace•L-Phe•0.5 D_2O	0.8 ± 0.5 /4
		L-Phe•0 D_2O	1.0 ± 0.3 /5
**Ace/D-Phe**	LCP	Ace•D-Phe•0.5 D_2O	1.1 ± 0.3 /5
		D-Phe•0 D_2O	0.9 ± 0.2 /5
	RCP	Ace•D-Phe•0.5 D_2O	0.8 ± 0.1 /5
		D-Phe•0 D_2O	1.0 ± 0.1 /5

References

(S1) Mohan, R.; Koo, K.-K.; Strege, C.; Myerson, A. S. Effect of Additives on the Transformation Behavior of L-Phenylalanine in Aqueous Solution. *Industrial Engineering Chemistry Research* **2001**, *40*, 6111–6117. DOI: 10.1021/ie0105223.

(S2) Ito, S.; Sugiyama, T.; Toitani, N.; Katayama, G.; Miyasaka, H. Application of Fluorescence Correlation Spectroscopy to the Measurement of Local Temperature in Solutions under Optical Trapping Conditions. The *Journal of Physical Chemistry B* **2007**, *111*, 2365–2371. DOI: 10.1021/jp065156l.

(S3) Simon, A.; Libchaber, A. Escape and Synchronization of a Brownian Particle. *Physical Review Letters* **1992**, *68*, 3375–3378. DOI: 10.1103/PhysRevLett.68.3375.

(S4) Kramers, H. A. Brownian Motion in a Field of Force and the Diffusion Model of Chemical Reactions. *Physica VII* **1940**, *7*, 284–304. DOI: 10.1016/S0031-8914(40)90098-2.

(S5) Gowayed, O. Y.; Moosa, T.; Moratos, A. M.; Hua, T.; Arnold, S.; Garetz, B. A. Dynamic Light Scattering Study of a Laser-Induced Phase-Separated Droplet of Aqueous Glycine. *Journal of Physical Chemistry B* **2021**, *125*, 7828–7839. DOI: 10.1021/acs.jpcb.1c02620.
